# Rhinovirus remains prevalent in school teenagers during fight against COVID‐19 pandemic

**DOI:** 10.1002/iid3.381

**Published:** 2020-11-28

**Authors:** Di Wu, Jianyun Lu, Zhangyu Sun, Lan Cao, Qing Zeng, Qun Liu, Tiantian Wu, Zhicong Yang

**Affiliations:** ^1^ Department for Infectious Diseases Control and Prevention Guangzhou Center for Disease Control and Prevention Guangzhou China; ^2^ Department of Respiratory Medicine Guangzhou Panyu Central Hospital Guangzhou China; ^3^ Department of Virology and Immunology Guangzhou Center for Disease Control and Prevention Guangzhou China; ^4^ Pharmacology Laboratory Guangdong Provincial Institute of Biological Products and Materia Medica Guangzhou China; ^5^ Institute of Human Virology Zhongshan School of Medicine, Sun Yat‐sen University Guangzhou China

**Keywords:** rhinovirus, HRV, SARS‐COV‐2, COVID‐19

The coronavirus disease 2019 (COVID‐19) caused by the severe acute respiratory syndrome coronavirus 2 is now being a disaster, as of November 3, 2020, the World Health Organization have reported 46 million cases and 1.2 million deaths globally.^1^ Studies have reported that respiratory infectious diseases such as influenza, varicella, herpes zoster, rubella, and measles are suppressed during the fight against the pandemic of COVID‐19,^2^, ^3^, ^4^ and the respiratory infection.^5^ However, while schools in Guangzhou have been reopened, we have monitored several outbreaks of unknown fever, and later proved to be human rhinovirus (HRV) infections.

We have collected the outbreak data from the field epidemiological investigation, the basic information and clinical characteristics were collected using EXCEL, and all throat swabs were collected during the investigation. All data generated or analyzed during the study were included in this manuscript.

The throat swabs were tested for influenza A virus, influenza A (H1) virus, influenza A (H3) virus, influenza A (H1N1) pdm2009 virus, influenza B virus, respiratory syncytial virus, parainfluenza virus (Type Ⅰ, Ⅱ, Ⅲ, and Ⅳ), bocavirus, human metapneumovirus, HRV, adenovirus, human coronavirus (OC43, 229E, HKU‐1, and NL63), Legionella, *Mycoplasma pneumoniae*, and *Chlamydia pneumoniae*, using the NxTAG Respiratory Pathogen Panel (Luminex, Lot: XK051C‐1045) according to the manufacturer's protocol.

All the specimens were negative for the viruses listed in the method but the rhinovirus. A total of 61 samples were collected and 17 (27.87%) of which were positive for rhinovirus (Table 1). We next conducted a *χ* ^2^ test and found that the rate of patients with runny nose was statistically higher in the HRV positive patients than that of in the HRV‐negative patients (Table 2). It is worth noting that a 14‐year‐old girl had recorded with diarrhea.

**Table 1:** The clinical characteristics of the tested patients in the five schools

Characteristics		School A	School B	School C	School D	School E	Total
No. of total patients		14	16	21	16	55	122
No. of total students		1,483	1,119	2,007	1,756	1,623	
Attack rate (%)		0.94	1.43	1.05	0.91	3.39	
Fever patients^a^		14	2	9	0	62	
Sampling patients		12	14	12	11	12	61
Sex							
Male		4	8	7	11	3	33
Female		8	6	5	0	9	28
Cough							
Yes		3	7	4	9	1	24
No		9	7	8	2	11	37
Sore throat							
Yes		8	9	8	1	6	32
No		4	5	4	10	6	29
Headache							
Yes		3	1	2	1	0	7
No		9	13	10	10	12	54
Runny nose							
Yes		9	9	8	8	4	38
No		3	5	4	3	8	23
Sneeze							
Yes		0	5	3	0	3	11
No		12	9	9	11	9	50
Human rhinovirus test							
Positive		3	1	4	6	3	17
Negative		9	13	8	5	9	44

^a^ The patients with temperature ≥ 37.3°C are defined as having fever.

**Table 2:** The characteristics of all the patients in the five schools

Clinical characteristics		No. of samples	HRV test			Total	HRV^a^	
			−	+	Total		*χ* ^2^	*p*
Age (average)		9.7	11.5	11.1	11.2	10.5		
Sex								
Male		37	8	25	33	70	0.470	.493
Female		24	9	19	28	52		
Fever^b^								
Yes		44	6	21	27	71	0.786	.381
No		17	11	23	34	51		
Cough								
Yes		24	7	17	24	48	0.033	.856
No		37	10	27	37	74		
Sore throat								
Yes		27	8	24	32	59	0.276	.6
No		34	9	20	29	63		
Headache								
Yes		3	1	6	7	10	0.726	.394
No		58	16	38	54	112		
Runny Nose								
Yes		27	14	24	38	65	**4.037**	**.045**
No		34	3	20	23	57		
Sneeze								
Yes		6	3	8	11	17	0.002	.961
No		55	14	36	50	105		

^a^ χ2 test of the human rhinovirus (HRV) positive and negative patients.

^b^ The patients with temperature ≥ 37.3°C are defined as having fever.

Most “common colds” were caused by HRV, which was reported to be the most common pathogen of upper respiratory tract infection, and also a common copathogen in patients with respiratory tract infection.^6^ An investigation of 452 children with acute respiratory infection reported that acute respiratory infection in children was mainly infected by HRV (11.28%), and most of the HRV infection occurred was in winter.^7^ Respiratory virus surveillance studies showed that the positive rate of HRV was 16.4%–35.1%.^8^, ^9^, ^10^, ^11^, ^12^, ^13^ During the COVID‐19 pandemic, the government had taken all possible means to stop the pandemic, and successfully stopped the COVID‐19 pandemic in Guangzhou, and the common respiratory infectious diseases, such as influenza and pneumonia as well.^3^ As expected, the HRV should be controlled as a respiratory infectious disease; however, the HRV outbreaks are still being reported in Guangzhou. Leung et al.^14^ came with an analogical conclusion: they found that there were no significant differences in the viral load of the with or without the face masks in patients infected with HRV. A study^15^ conducted in Shanghai, China, found that the positive rate of HRV among influenza‐like illness patients was 6.46%, mainly reported in children under 10 years old, and the HRV‐A was the main subtype. Perry Markovich et al.^16^ reported that the upper respiratory infection (URI) was observed increased in preschool and primary school‐age children while they back to school in new school year in Israel. The study showed that the URI cases were more likely to be detected positive for HRV and other viruses than that of the health controls, and the health controls also could be detected positive for HRV, suggests that there are carriers of HRV in healthy adolescents, so the control measures such as lockdown of the city and closure of the public places have lowered the risk of the respiratory infectious diseases, but when the schools reopened, the students gathered together and increased the risk of HRV transmission, which raises the importance of epidemiological surveillance of other respiratory viruses during a pandemic.

## CONFLICT OF INTERESTS

The authors declare that there are no conflict of interests.

## AUTHOR CONTRIBUTIONS

*Concept and design*: Di Wu, Jianyun Lu, and Zhangyu Sun. *Acquisition, analysis, or interpretation of data*: Qun Liu and Tiantian Wu. *Drafting of the manuscript*: Di Wu and Qun Liu. *Experiment*: Lan Cao and Qing Zeng. *Statistical analysis*: Di Wu. *Supervision*: Zhicong Yang.
